# *N*-Phenyl-*N*-(prop-2-en-1-yl)benzene­sulfonamide

**DOI:** 10.1107/S1600536810013152

**Published:** 2010-04-14

**Authors:** Islam Ullah Khan, Gui-Ying Dong, Sharafat Ali, Shahzad Sharif, Zeeshan Haide

**Affiliations:** a Materials Chemistry Laboratory, Department of Chemistry, Government College University, Lahore 54000, Pakistan; b College of Chemical Engineering and Biotechnology, Hebei Polytechnic University, Tangshan 063009, People’s Republic of China; c HEJ Research Institute of Chemistry, University of Karachi, Pakistan

## Abstract

In the mol­ecule of the title compound, C_15_H_15_NO_2_S, the dihedral angle between the two phenyl rings is 41.8 (3)°. The S atom has a distorted tetra­hedral environment. In the crystal structure, C—H⋯O hydrogen bonds link the molecules into a ribbon-like structure along [010].

## Related literature

For details of the biological activity and pharmaceutical applications of sulfonamide derivatives, see: Kazmierski *et al.* (2004 ▶); Beate *et al.* (1998 ▶); Skrzipczyk *et al.* (1994 ▶). For related structures, see: Arshad *et al.* (2009 ▶); Khan *et al.* (2009 ▶).
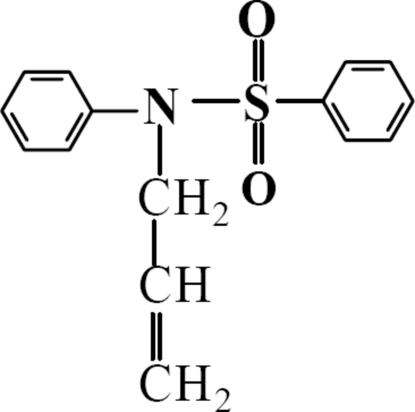


## Experimental

### 

#### Crystal data

C_15_H_15_NO_2_S
*M* *_r_* = 273.35
Monoclinic, 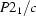

*a* = 11.6302 (8) Å
*b* = 5.7041 (4) Å
*c* = 21.9408 (14) Å
β = 103.535 (4)°
*V* = 1415.12 (17) Å^3^
*Z* = 4
Mo *K*α radiation
μ = 0.23 mm^−1^
*T* = 295 K
0.25 × 0.12 × 0.08 mm

#### Data collection

Bruker SMART CCD area-detector diffractometer
Absorption correction: multi-scan (*SADABS*; Sheldrick, 1996 ▶) *T*_min_ = 0.958, *T*_max_ = 0.978
9448 measured reflections
2467 independent reflections
1803 reflections with *I* > 2σ(*I*)
*R*_int_ = 0.046

#### Refinement

*R*[*F* ^2^ > 2σ(*F* ^2^)] = 0.060
*wR*(*F* ^2^) = 0.245
*S* = 0.93
2467 reflections
172 parameters
H-atom parameters constrained
Δρ_max_ = 0.47 e Å^−3^
Δρ_min_ = −0.58 e Å^−3^

### 

Data collection: *SMART* (Bruker, 1998 ▶); cell refinement: *SAINT* (Bruker, 1999 ▶); data reduction: *SAINT*; program(s) used to solve structure: *SHELXS97* (Sheldrick, 2008 ▶); program(s) used to refine structure: *SHELXL97* (Sheldrick, 2008 ▶); molecular graphics: *SHELXTL* (Sheldrick, 2008 ▶); software used to prepare material for publication: *SHELXTL*.

## Supplementary Material

Crystal structure: contains datablocks I, global. DOI: 10.1107/S1600536810013152/ci5071sup1.cif

Structure factors: contains datablocks I. DOI: 10.1107/S1600536810013152/ci5071Isup2.hkl

Additional supplementary materials:  crystallographic information; 3D view; checkCIF report

## Figures and Tables

**Table 1:** Hydrogen-bond geometry (Å, °)

*D*—H⋯*A*	*D*—H	H⋯*A*	*D*⋯*A*	*D*—H⋯*A*
C1—H1*A*⋯O1^i^	0.97	2.57	3.427 (6)	147
C5—H5⋯O2^ii^	0.93	2.39	3.307 (6)	167
C15—H15⋯O1^i^	0.93	2.54	3.415 (6)	158

Symmetry codes: (i) 
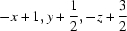
; (ii) 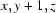.

**Fig. 1.**
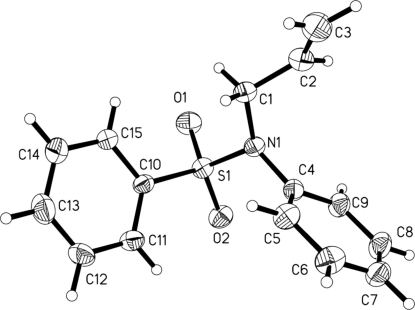
The molecular structure of the title compound, showing the atomic numbering and 30% probability displacement ellipsoids.

**Fig. 2.**
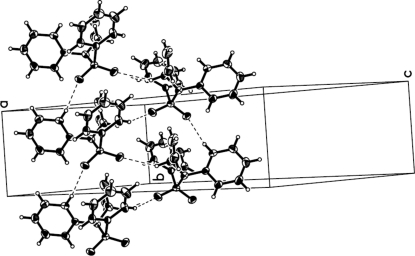
A partial packing diagram of the title compound. Hydrogen bonds are shown as dashed lines

## supplementary crystallographic information

### Comment

Benzenesulfonamide derivatives have been used as starting materials for the preparation of a variety of sulfonamide drugs, such as inhibitors of HIV infection (Kazmierski *et al.*, 2004) and antihypertensive drugs (Beate *et al.*, 1998). In addition, they have also been employed in the preparation of gene probe labelling (Skrzipczyk *et al.*, 1994). As an extension of our previous studies (Arshad *et al.*, 2009; Khan *et al.*, 2009), we report here the crystal structure of the title compound.

The molecular structure of the title compound, (I), is illustrated in Fig. 1. The dihedral angle between the two phenyl rings is 41.8 (3)°. Atom S1 has a distorted tetrahedral environment, with a O1—S1—O2 angle of 120.2 (2)°. The C10—S1—N1—C4 torsion angle in the central part of the molecule is 86.2 (3)°.

In the crystal structure, adjacent molecules are linked *via* C—H···O hydrogen bonds (Table 1) to form a ribbon-like structure along the *b* axis (Fig.2).

### Experimental

A mixture of *N*-phenyl benzenesulfonamide (0.5 g, 2.1552 mmol), sodium hydride (0.2 g, 8.333 mmol) and N,N-dimethylformamide (10 ml) was stirred at room temperature for 30 min and then allyl bromide (0.37 ml, 2.1552 mmol) was added. The stirring was continued further for a period of 3 h and the contents were poured over crushed ice. The precipitated product was isolated, washed and recrystallized from methanol solution.

### Refinement

H atoms were placed in calculated positions, with C–H = 0.93 or 0.97 Å and included in the final cycles of refinement using a riding model, with *U*_iso_(H) = 1.2*U*_eq_(parent atom).

### Crystal data

**Table:**

C_15_H_15_NO_2_S	*F*(000) = 576
*M**_r_* = 273.35	*D*_x_ = 1.283 Mg m^−^^3^
Monoclinic, *P*2_1_/*c*	Mo *K*α radiation, λ = 0.71073 Å
Hall symbol: -P 2ybc	Cell parameters from 3368 reflections
*a* = 11.6302 (8) Å	θ = 2.4–22.3°
*b* = 5.7041 (4) Å	µ = 0.23 mm^−^^1^
*c* = 21.9408 (14) Å	*T* = 295 K
β = 103.535 (4)°	Plate, colourless
*V* = 1415.12 (17) Å^3^	0.25 × 0.12 × 0.08 mm
*Z* = 4	

### Data collection

**Table:**

Bruker SMART CCD area-detector diffractometer	2467 independent reflections
Radiation source: fine-focus sealed tube	1803 reflections with *I* > 2σ(*I*)
graphite	*R*_int_ = 0.046
φ and ω scans	θ_max_ = 25.0°, θ_min_ = 1.8°
Absorption correction: multi-scan (*SADABS*; Sheldrick, 1996)	*h* = −13→13
*T*_min_ = 0.958, *T*_max_ = 0.978	*k* = −6→5
9448 measured reflections	*l* = −26→26

### Refinement

**Table:**

Refinement on *F*^2^	Primary atom site location: structure-invariant direct methods
Least-squares matrix: full	Secondary atom site location: difference Fourier map
*R*[*F*^2^ > 2σ(*F*^2^)] = 0.060	Hydrogen site location: inferred from neighbouring sites
*wR*(*F*^2^) = 0.245	H-atom parameters constrained
*S* = 0.93	*w* = 1/[σ^2^(*F*_o_^2^) + (0.1687*P*)^2^ + 2.1422*P*] where *P* = (*F*_o_^2^ + 2*F*_c_^2^)/3
2467 reflections	(Δ/σ)_max_ = 0.001
172 parameters	Δρ_max_ = 0.47 e Å^−^^3^
0 restraints	Δρ_min_ = −0.58 e Å^−^^3^

### Special details

**Table:**

Geometry. All esds (except the esd in the dihedral angle between two l.s. planes) are estimated using the full covariance matrix. The cell esds are taken into account individually in the estimation of esds in distances, angles and torsion angles; correlations between esds in cell parameters are only used when they are defined by crystal symmetry. An approximate (isotropic) treatment of cell esds is used for estimating esds involving l.s. planes.
Refinement. Refinement of F^2^ against ALL reflections. The weighted R-factor wR and goodness of fit S are based on F^2^, conventional R-factors R are based on F, with F set to zero for negative F^2^. The threshold expression of F^2^ > 2sigma(F^2^) is used only for calculating R-factors(gt) etc. and is not relevant to the choice of reflections for refinement. R-factors based on F^2^ are statistically about twice as large as those based on F, and R- factors based on ALL data will be even larger.

### Fractional atomic coordinates and isotropic or equivalent isotropic displacement parameters (Å^2^)

**Table:**

	*x*	*y*	*z*	*U*_iso_*/*U*_eq_	
S1	0.69888 (9)	−0.0377 (2)	0.71607 (5)	0.0425 (4)	
N1	0.6478 (3)	0.1073 (6)	0.65082 (15)	0.0421 (9)	
C9	0.7280 (4)	−0.0195 (9)	0.5639 (2)	0.0500 (11)	
H9	0.6824	−0.1545	0.5615	0.060*	
C10	0.7692 (3)	0.1656 (8)	0.77270 (17)	0.0398 (10)	
C13	0.8729 (5)	0.5034 (11)	0.8566 (2)	0.0683 (15)	
H13	0.9082	0.6188	0.8848	0.082*	
C3	0.4412 (6)	0.5309 (14)	0.5687 (3)	0.092 (2)	
H3A	0.4626	0.6644	0.5930	0.111*	
H3B	0.3918	0.5436	0.5288	0.111*	
C4	0.7260 (4)	0.1466 (8)	0.60926 (17)	0.0407 (10)	
C1	0.5584 (4)	0.2901 (9)	0.6524 (2)	0.0504 (11)	
H1A	0.5114	0.2434	0.6815	0.060*	
H1B	0.5982	0.4356	0.6675	0.060*	
C11	0.8892 (4)	0.2012 (9)	0.7843 (2)	0.0525 (12)	
H11	0.9351	0.1128	0.7633	0.063*	
C5	0.7939 (4)	0.3460 (8)	0.6128 (2)	0.0512 (11)	
H5	0.7929	0.4574	0.6437	0.061*	
C2	0.4799 (4)	0.3294 (11)	0.5901 (2)	0.0631 (14)	
H2	0.4569	0.1994	0.5645	0.076*	
C15	0.7010 (4)	0.2992 (10)	0.80332 (19)	0.0548 (13)	
H15	0.6199	0.2745	0.7958	0.066*	
C7	0.8644 (4)	0.2144 (11)	0.5249 (2)	0.0616 (14)	
H7	0.9106	0.2384	0.4962	0.074*	
C6	0.8631 (5)	0.3801 (10)	0.5706 (2)	0.0616 (13)	
H6	0.9090	0.5148	0.5728	0.074*	
C14	0.7545 (5)	0.4699 (11)	0.8452 (2)	0.0686 (16)	
H14	0.7090	0.5619	0.8655	0.082*	
O2	0.7865 (3)	−0.1951 (6)	0.70389 (14)	0.0545 (9)	
O1	0.5986 (3)	−0.1260 (6)	0.73525 (15)	0.0582 (9)	
C8	0.7983 (5)	0.0159 (10)	0.5219 (2)	0.0616 (14)	
H8	0.8005	−0.0963	0.4914	0.074*	
C12	0.9411 (4)	0.3693 (11)	0.8272 (2)	0.0641 (15)	
H12	1.0226	0.3915	0.8361	0.077*	

### Atomic displacement parameters (Å^2^)

**Table:**

	*U*^11^	*U*^22^	*U*^33^	*U*^12^	*U*^13^	*U*^23^
S1	0.0455 (7)	0.0354 (7)	0.0488 (7)	0.0000 (5)	0.0153 (5)	0.0014 (4)
N1	0.0414 (18)	0.042 (2)	0.0431 (18)	0.0055 (16)	0.0106 (14)	−0.0030 (16)
C9	0.057 (3)	0.046 (3)	0.047 (2)	−0.001 (2)	0.014 (2)	−0.011 (2)
C10	0.037 (2)	0.044 (3)	0.041 (2)	0.0036 (18)	0.0143 (16)	0.0044 (18)
C13	0.070 (4)	0.075 (4)	0.055 (3)	−0.012 (3)	0.005 (2)	−0.016 (3)
C3	0.096 (5)	0.112 (6)	0.070 (4)	0.050 (4)	0.020 (3)	0.021 (4)
C4	0.045 (2)	0.039 (2)	0.037 (2)	0.0094 (19)	0.0076 (16)	0.0005 (17)
C1	0.044 (2)	0.057 (3)	0.051 (2)	0.010 (2)	0.0128 (18)	−0.005 (2)
C11	0.040 (2)	0.064 (3)	0.056 (3)	0.000 (2)	0.0167 (19)	0.002 (2)
C5	0.059 (3)	0.036 (3)	0.061 (3)	0.005 (2)	0.019 (2)	−0.003 (2)
C2	0.052 (3)	0.079 (4)	0.057 (3)	0.016 (3)	0.010 (2)	0.002 (3)
C15	0.039 (2)	0.080 (4)	0.045 (2)	0.006 (2)	0.0099 (18)	−0.011 (2)
C7	0.058 (3)	0.078 (4)	0.053 (3)	0.014 (3)	0.022 (2)	0.015 (3)
C6	0.066 (3)	0.049 (3)	0.074 (3)	0.002 (3)	0.026 (3)	0.013 (3)
C14	0.062 (3)	0.086 (4)	0.056 (3)	0.013 (3)	0.008 (2)	−0.022 (3)
O2	0.0622 (19)	0.0412 (19)	0.0631 (19)	0.0148 (15)	0.0206 (15)	0.0029 (15)
O1	0.0529 (18)	0.055 (2)	0.070 (2)	−0.0140 (16)	0.0216 (15)	0.0053 (17)
C8	0.074 (3)	0.065 (4)	0.050 (3)	0.012 (3)	0.022 (2)	−0.008 (2)
C12	0.039 (2)	0.091 (4)	0.061 (3)	−0.018 (3)	0.009 (2)	−0.003 (3)

### Geometric parameters (Å, °)

**Table:**

S1—O1	1.422 (3)	C1—C2	1.473 (6)
S1—O2	1.429 (3)	C1—H1A	0.97
S1—N1	1.640 (3)	C1—H1B	0.97
S1—C10	1.756 (4)	C11—C12	1.380 (7)
N1—C4	1.448 (5)	C11—H11	0.93
N1—C1	1.479 (5)	C5—C6	1.377 (7)
C9—C4	1.379 (6)	C5—H5	0.93
C9—C8	1.382 (7)	C2—H2	0.93
C9—H9	0.93	C15—C14	1.382 (7)
C10—C11	1.374 (6)	C15—H15	0.93
C10—C15	1.382 (6)	C7—C8	1.361 (8)
C13—C14	1.354 (8)	C7—C6	1.379 (7)
C13—C12	1.368 (8)	C7—H7	0.93
C13—H13	0.93	C6—H6	0.93
C3—C2	1.283 (8)	C14—H14	0.93
C3—H3A	0.93	C8—H8	0.93
C3—H3B	0.93	C12—H12	0.93
C4—C5	1.376 (6)		
			
O1—S1—O2	120.2 (2)	H1A—C1—H1B	107.9
O1—S1—N1	106.41 (19)	C10—C11—C12	119.4 (4)
O2—S1—N1	106.38 (18)	C10—C11—H11	120.3
O1—S1—C10	107.66 (19)	C12—C11—H11	120.3
O2—S1—C10	108.22 (19)	C4—C5—C6	119.8 (5)
N1—S1—C10	107.3 (2)	C4—C5—H5	120.1
C4—N1—C1	117.0 (3)	C6—C5—H5	120.1
C4—N1—S1	118.4 (3)	C3—C2—C1	124.3 (6)
C1—N1—S1	116.6 (3)	C3—C2—H2	117.8
C4—C9—C8	119.6 (5)	C1—C2—H2	117.8
C4—C9—H9	120.2	C14—C15—C10	119.4 (4)
C8—C9—H9	120.2	C14—C15—H15	120.3
C11—C10—C15	120.2 (4)	C10—C15—H15	120.3
C11—C10—S1	120.9 (3)	C8—C7—C6	120.2 (5)
C15—C10—S1	118.9 (3)	C8—C7—H7	119.9
C14—C13—C12	120.7 (5)	C6—C7—H7	119.9
C14—C13—H13	119.6	C5—C6—C7	120.0 (5)
C12—C13—H13	119.6	C5—C6—H6	120.0
C2—C3—H3A	120.0	C7—C6—H6	120.0
C2—C3—H3B	120.0	C13—C14—C15	120.1 (5)
H3A—C3—H3B	120.0	C13—C14—H14	119.9
C5—C4—C9	120.2 (4)	C15—C14—H14	119.9
C5—C4—N1	121.9 (4)	C7—C8—C9	120.3 (5)
C9—C4—N1	117.9 (4)	C7—C8—H8	119.9
C2—C1—N1	111.8 (4)	C9—C8—H8	119.9
C2—C1—H1A	109.3	C13—C12—C11	120.1 (4)
N1—C1—H1A	109.3	C13—C12—H12	120.0
C2—C1—H1B	109.3	C11—C12—H12	120.0
N1—C1—H1B	109.3		
			
O1—S1—N1—C4	−158.8 (3)	C4—N1—C1—C2	58.4 (5)
O2—S1—N1—C4	−29.5 (4)	S1—N1—C1—C2	−153.1 (4)
C10—S1—N1—C4	86.2 (3)	C15—C10—C11—C12	0.8 (7)
O1—S1—N1—C1	53.2 (4)	S1—C10—C11—C12	177.3 (4)
O2—S1—N1—C1	−177.5 (3)	C9—C4—C5—C6	0.5 (7)
C10—S1—N1—C1	−61.9 (3)	N1—C4—C5—C6	−177.3 (4)
O1—S1—C10—C11	150.3 (4)	N1—C1—C2—C3	−141.8 (6)
O2—S1—C10—C11	19.0 (4)	C11—C10—C15—C14	0.5 (7)
N1—S1—C10—C11	−95.5 (4)	S1—C10—C15—C14	−176.1 (4)
O1—S1—C10—C15	−33.1 (4)	C4—C5—C6—C7	−0.1 (7)
O2—S1—C10—C15	−164.5 (4)	C8—C7—C6—C5	−0.7 (8)
N1—S1—C10—C15	81.1 (4)	C12—C13—C14—C15	−0.1 (9)
C8—C9—C4—C5	−0.2 (7)	C10—C15—C14—C13	−0.9 (8)
C8—C9—C4—N1	177.7 (4)	C6—C7—C8—C9	1.0 (8)
C1—N1—C4—C5	55.6 (5)	C4—C9—C8—C7	−0.6 (7)
S1—N1—C4—C5	−92.3 (4)	C14—C13—C12—C11	1.5 (9)
C1—N1—C4—C9	−122.2 (4)	C10—C11—C12—C13	−1.8 (8)
S1—N1—C4—C9	89.8 (4)		

### Hydrogen-bond geometry (Å, °)

**Table:**

*D*—H···*A*	*D*—H	H···*A*	*D*···*A*	*D*—H···*A*
C1—H1A···O1^i^	0.97	2.57	3.427 (6)	147
C5—H5···O2^ii^	0.93	2.39	3.307 (6)	167
C15—H15···O1^i^	0.93	2.54	3.415 (6)	158

Symmetry codes: (i) −*x*+1, *y*+1/2, −*z*+3/2; (ii) *x*, *y*+1, *z*.
